# Antiproliferative Activities of Water Infusions from Leaves of Five *Cornus* L. Species

**DOI:** 10.3390/molecules201219786

**Published:** 2015-12-16

**Authors:** Vladimír Forman, Mária Haladová, Daniel Grančai, Mária Ficková

**Affiliations:** 1 Department of Pharmacognosy and Botany, Faculty of Pharmacy, Comenius University in Bratislava, Odbojárov 10, Bratislava 832 32, Slovakia; haladova.maria@gmail.com (M.H.); grancai@fpharm.uniba.sk (D.G.); 2 Laboratory of Cell Endocrinology, Institute of Experimental Endocrinology, Slovak Academy of Sciences, Vlárska 3, Bratislava 833 06, Slovakia; maria.fickova@savba.sk

**Keywords:** *Cornus*, antiproliferative activity, MCF-7 cells, flavonoids, THD, polyphenols, tannins

## Abstract

Cornaceae plants are known for their edible berries, and their leaves are used as tea. In the present study aqueous leaf extracts from *Cornus mas* (CM), *C. alba* (CA), *C. flaviramea* (CF), *C. kousa* (CK), and *C. officinalis* (CO) were tested for their antiproliferative activity in human breast cancer cells (MCF-7). Dose- (50–750 µg/mL) and time (24, 48, 72 h)-dependent antiproliferative effects were measured by WST-1, and correlated with the content of flavonoids (FL), total hydroxycinnamic derivatives (THD), total polyphenols (TP) and tannins (T). Extracts induced time dependent decreases in cell survival; CA, CO and CM were the most effective (11.2%, 10.3% and 11.1%, after 72 h). The ED_50_ (effective dose) values were similar for all extracts and times tested. The THD and TP were identical in all samples, while a two-fold higher T content was present in CK and CO, and of FL in CF. The maximal effects (% of surviving cells) negatively correlated with the T and TP levels, and positively with FL and THD. The results demonstrate the significant antiproliferative effects of the tested water extracts in MCF-7 cells, in which CA, CO and CM are the most effective; and the effectiveness is related to the T and TP contents.

## 1. Introduction

Despite the huge production and consumption of synthetic pharmaceuticals, natural products remain the main source of new therapeutic molecules. The plant material is extensively investigated with the aim to discover new cytostatic/antiproliferative active compounds.

Plants from the Cornaceae family are typically trees or shrubs. The genus *Cornus* is widespread, mostly in northern temperate climates [1]. The Slovak flora comprises six *Cornus* species (*C. mas*, *C. australis*, *C. sanguinea*, *C. hungarica*, *C. sericea*, *C. alba*) native to moist deciduous woodlands and the sunny slopes of highlands. Other species (e.g., *C. kousa*, *C. officinalis*, *C. florida*) are cultivated as ornamental plants or found in botanical gardens.

Some species of this family are used as parts of healing mixtures within Traditional Asian Medicines (Chinese, Korean). Extracts or particular herb parts (especially fruits) of several *Cornus* species have traditionally been used to treat colds, flu, urinary inflammation, diarrhoea, and to improve liver and kidney functions [2,3].

In Slovakia Cornelian cherries (*C. mas*) are used for the preparation of juices, syrups, and jams. Traditionally they were used for digestive disorders, fever and inflammation [4]. The alcoholic distillate *drienkovica* is also currently very popular.

Cornaceae (*C. mas*—fruits, *C. florida*—bark) also possess antimicrobial, antimalarial and antidiabetic properties [2,5]. Previous studies reported cytotoxic activity of some *Cornus* species extracts (*C. officinalis* fruits—aqueous, ethanolic extracts, *C. amomum*). *In vitro* antiproliferative effects of these extracts were described for several cell line types (HT29, HCT116—colon cancer, MCF-7, MDA-MB-231—breast cancer) [6,7,8]. Antiproliferative activity was described for some Cornaceae-isolated pure compounds—morroniside (*C. officinalis*—fruits), betulinic acid derivatives and other non-polar compounds (*C. florida*—bark), and anthocyanins, such as delphinidin 3-*O*-glucoside and delphinidin 3-*O*-rutinoside (*C. alternifolia, C. controversa*—fruits) [5,9,10].

In the present study we investigated the antiproliferative activity of aqueous extracts of the leaves of five *Cornus* species. The effects were correlated with the content of bioactive secondary metabolites: flavonoids, total hydroxycinnamic derivatives, total polyphenols and tannins.

## 2. Results and Discussion

**Figure 1:**
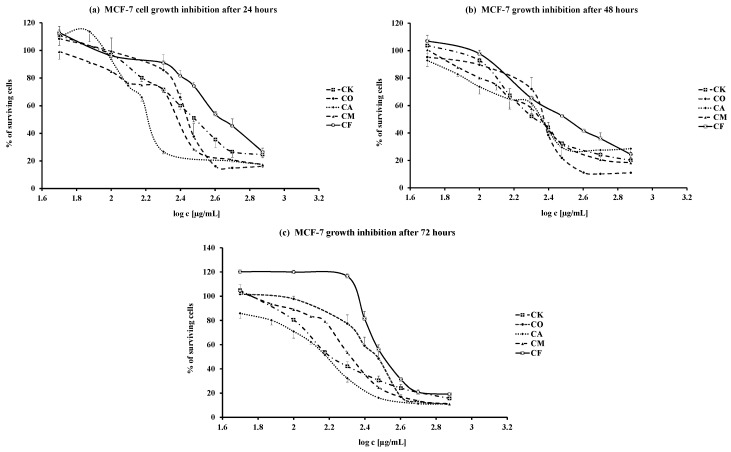
(**a**–**c**) Time and dose dependent MCF-7 cells growth inhibition after 24(**a**); 48(**b**); and 72(**c**) hours treatment with leaves water extracts from *Cornus* species (CA—*Cornus alba*, CO—*C. officinalis*, CK—*C. kousa*, CM—*C. mas*, CF—*C. flaviramea*).

For the cell growth analyses we preferred to use the WST-1 cell proliferation reagent (Roche, Mannheim, Germany), as the solubility of the active compound is advantageous over MTT. The results of dose- and time-dependent effects of the five tested leaves water extracts from *Cornus* species are illustrated by the respective dose response curves shown in Figure 1. The percentage of surviving cells, as the effect of maximal dose, is presented in Table 1. The table clearly demonstrates that a marked decline of living cells is already observable even after 24 h and the cell growth gradually declines over the period of 72 h. The most potent were CO, CM and CA leaves water extracts with the lowest % of surviving cells after 72 h: 10.3% ± 0.5% (CO), 11.1% ± 0.3% (CM) and 11.2% ± 0.2% (CA), respectively. The values are two-fold lower than that for CF (19.2% ± 0.3%).

**Table 1:** Maximal dose (750 μg·mL^−1^) effect on MCF-7 cells growth inhibition (% of survived cells) induced by leaves water extracts of five *Cornus* species after treatment for 24, 48 and 72 h.

Sample	24 h	48 h	72 h
CA	17.5 ± 0.3 ^a,b,d^	18.9 ± 0.1	11.2 ± 0.2
CO	16.1 ± 0.4 ^c,e^	10.4 ± 0.3 ^1,2^	10.3 ± 0.5
CK	24.7 ± 2.4 ^a,c,^*	20.1 ± 1.8 ^1^	15.6 ± 0.8 *
CM	17.5 ± 0.1	18.3 ± 0.4	11.1 ± 0.3
CF	26.6 ± 2.7 ^b,d,e^	24.4 ± 1.6 ^2^	19.2 ± 0.3

The values are expressed as mean ± SE of three individual experiments performed in triplicates for each dose. Statistical differences between individual extracts/time groups are labeled with the same asterisk: ^a,b^ (*p* < 0.05), ^c,d,1,^* (*p* < 0.01), ^e,2^ (*p* < 0.001). CA—*Cornus alba*, CO—*C. officinalis*, CK—*C. kousa*, CM—*C. mas*, CF—*C. flaviramea*.

The biological activity of plant material is generally related to the content of components that are soluble either in polar, non-polar or semipolar solvents. Here we present the content of water soluble secondary metabolites in leaves. Table 2 shows substantial differences in the abundances of the determined substances between individual *Cornus* species.

The contents of flavonoids (expressed as hyperoside), THD—total hydroxycinnamic derivatives (expressed as rosmarinic acid), total polyphenols and tannins (expressed as pyrogallol) were analysed as described in details in the Experimental Section.

The highest flavonoids content was detected in CF (CF > CO = CK > CA > CM) together with the lowest presence of total polyphenols (CF < CA = CM < CK = CO). The same sequence of plant species was observed for the content of tannins. The amounts of hydroxycinnamic acid derivatives were almost the same with no differences between each species.

Next we investigated the relation between the content of individual secondary metabolites and the maximal antiproliferative effect (e.g., the % of surviving cells). The correlation coefficients are listed in Table 3.

**Table 2:** Content (w%) of secondary metabolites in aqueous leaves extracts of five *Cornus* species.

Sample	Flavonoids	THD	Total Polyphenols	Tannins
CA	0.28 ± 0.04 ^d^	1.1 ± 0.04	8.1 ± 0.13 ^c^	4.8 ± 0.12 ^b,d^
CO	0.37 ± 0.03 ^b,^*	1.4 ± 0.10	10.1 ± 0.82 ^b^	7.4 ± 0.76 ^b,c^
CK	0.36 ± 0.006 ^a^	1.3 ± 0.02	9.6 ± 0.80 ^a^	7.2 ± 0.67 ^a,d^
CM	0.21 ± 0.01 ^c,^*	1.2 ± 0.01	8.3 ± 0.11 ^d^	5.8 ± 0.10 ^e^
CF	0.72 ± 0.04 ^a,b,c,d^	1.5 ± 0.10	6.1 ± 0.20 ^a,b,c,d^	3.5 ± 0.14 ^a,c,e^

The values are expressed as mean ± SE of three parallel analyses of each secondary metabolite. Statistical differences between individual extracts and metabolite are labeled with the same asterisk: ^a,b,c,d^ (*p* < 0.001), * (*p* < 0.05) = for flavonoids; ^a,b^ (*p* < 0.001), ^c,d^ (*p* < 0.05) = for total polyphenols; ^a,b,c^ (*p* < 0.001), ^d,e^ (*p* < 0.01) = for tannins. CA—*Cornus alba*, CO—*C. officinalis*, CK—*C. kousa*, CM—*C. mas*, CF—*C. flaviramea*, THD—total hydroxycinnamic derivatives.

**Table 3:** Correlation coefficients (R) and statistical signifficance (*p*<) for the relationship between max. effect (% of cell survival) and the content of secondary metabolites.

Secondary Metabolite Content	24 h		48 h		72 h	
	R	*p*<	R	*p*<	R	*p*<
Flavonoids	0.9921	0.01	0.9999	0.001	0.9928	0.01
THD	0.9704	0.05	0.9894	0.02	0.9266	0.05
Tannins	−0.9537	0.05	−0.9874	0.02	−0.9311	NS
Polyphenols	−0.9072	0.05	−0.9916	0.01	−0.9998	0.001

THD—total hydroxycinnamic derivatives; NS = non–significant. The data were calculated from results presented in Table 1 and Table 2.

The negative correlation coefficients for tannins and total polyphenols indicate that the antiproliferative effect increases with increased content for almost all time exposures. The statistically significant positive correlation between flavonoids and hydroxycinnamic acid derivatives content and the biological effect (% of surviving cells) means that these metabolites protect MCF-7 cells from destruction and support cell proliferation during 72 h exposure.

Literature sources reveal a lot of phytochemical and pharmacological information about Cornaceae fruits used in Asian and Eastern European countries. Comparatively less information is available on the leaves and their pharmacology and chemistry. Earlier phytochemical studies of Cornaceae leaves revealed the presence of flavonoids, tannins, other polyphenolic derivatives and iridoids [11,12,13]. Previous pharmacological studies also demonstrated cytotoxic (*C. officinalis*—fruits) [8] and the effects, such as antiparasitic and antimicrobial (*C. florida*—bark) [5], antioxidative (*C. mas*—fruits) [14], antidiabetic (*C. officinalis*—fruits) [15].

The antioxidative effect, modulation of critical enzymes, induction of apoptosis and antibody treatment are fundamental for cancer chemotherapy. Anticancer activity as well as the ability of some polyphenols to reduce or inhibit DNA damage is well documented, e.g., polyphenols activating tumor necrosis factor-related apoptosis-inducing ligand (TRAIL) [16], or gallic acid as an inductor of selective cell death in cancer cells [17].

Our results obtained with Cornaceae leaves extracts extend a previous study with fruits of *Cornus officinalis* [18]. The authors showed the ability of the extract to significantly reduce cell growth of MCF-7 (human breast cancer cells) in dose- and time-dependent manners. The extract (concentration of 500 µg·mL^−1^) inhibited the cell growth more than 60% after 24, 48 and 72 h of treatment. Despite using as different type of extract and herb part, it can be stated that our results obtained with aqueous leaves extracts displayed similar antiproliferative effects as described above. The antiproliferative properties of *C. mas*, *C. officinalis*, *C. kousa*, *C. alba* and *C. flaviramea* aqueous leaves extracts and their relation to the polar substances content presented in our study call for detailed analyses in other cancer cell lines and of the intracellular molecular mechanisms of cell growth inhibition.

## 3. Experimental Section

### 3.1. Plant Material

Leaves of *Cornus mas* (CM), *C. alba* (CA), *C. flaviramea* (CF), *C. kousa* (CK), and *C. officinalis* (CO) were collected at the Tesárske Mlyňany Arboretum (Institute of Forest Ecology, Slovak Academy of Sciences, Slovakia) in October 2014. Botanical identification was performed by Ing. Peter Hoťka (Mlyňany Arboretum). Voucher specimens have been deposited at the Department of Pharmacognosy and Botany (Bratislava, Slovakia) (CM-2014, CA-2014, CF-2014, CK-2014, CO-2014).

### 3.2. Extract Preparation

Harvested leaves were dried for 5 days at room temperature and milled in a laboratory mill. Particle size was adjusted using a No. 355 sieve (European Pharmacopoeia, “Particle-size distribution estimation by analytical sieving”) [19]. Water infusions were prepared according to the article “Decocta. Infusa.” In brief: leaves (10 g) were extracted in boiling water (100 mL) for 5 min and then the infusion was cooled at room temperature for 45 min, filtered through cotton wool and dried by freeze-drying [20]. The yields of individual extracts were (w%): CM—55, CA—20, CF—50, CK—20 and CO—32, respectively. The method is one generally used in our laboratories and was published previously [21].

### 3.3. Secondary Metabolites Quantification

Methods as well as chemicals used for individual groups of secondary metabolites quantification are in accordance with particular monographs of the European Pharmacopoeia 8th edition, and are briefly described below. All colorimetric measurements were performed on a GENESYS™ 10 spectrophotometer (Thermo Electron Corporation, Cambridge, UK). All the chemicals used were of analytical grade.

#### 3.3.1. Total Polyphenols and Tannins Spectrophotometric Assay

Total polyphenols were measured as the complex with phosphomolybdotungstic reagent, absorbance at 760 nm according to the monograph “Hamamelidis folium” [22]. Tannins were determined as the difference of total polyphenols and polyphenols not adsorbed by hide powder and measured at 760 nm. The content (%) of total polyphenols and tannins was expressed as pyrogallol (reference compound).

#### 3.3.2. Flavonoids Spectrophotometric Assay

The extract was prepared according to the monograph “Betulae folium” [23]. Acetone extract of the powdered material (20 mL) was diluted with water (20 mL) and further was extracted (liquid-liquid) with ethyl acetate (1 × 15 mL, 3 × 10 mL) to obtain an ethyl acetate extract stock solution containing only flavonoids. The absorbance of the complex of the flavonoids with aluminium chloride reagent was measured at 425 nm and expressed as hyperoside (reference compound).

#### 3.3.3. Hydroxycinnamic Derivatives Spectrophotometric Assay

An ethanolic extract was prepared in accordance with the monograph “Rosmarini folium” [24] and used for the reaction with Arnow’s reagent. The final coloured product's absorbance was measured at 505 nm (using rosmarinic acid as the reference).

### 3.4. Cell Culture and Treatment

Human breast cancer epithelial cell line MCF-7 was obtained from Prof. G. Cremel (INSERM U682, Strasbourg, France). The cells were grown and passaged routinely as monolayer cultures in 75 cm^2^ flasks (Sarstedt, Nümbrecht, Germany). In our experiments, the cells were used at passages 10–25. In proliferation studies MCF-7 cells were seeded into 96-well plates at a density 1 × 10^4^/well in Dulbecco’s modified Eagle’s medium (DMEM) supplemented with 10% fetal bovine serum (FBS), both from Gibco BRL (Thermo Fisher Scientific, Waltham, MA, USA), antibiotics (penicillin 20,000 U/L, streptomycin 20,000 U/L, and gentamycin 80 mg/L, Cambrex, East Rutherford, NJ, USA) and cultivated in a humified atmosphere of 5% CO_2_ and 95% air at 37 °C. After 24 h of attachment, the medium was changed for fresh one and the tested extracts were added in various concentrations (50–750 µg/mL) for construction of dose response curves. The experiments with individual extracts were repeated three times with each dose in triplicates. Reference cells (control values) were cultivated in culture medium alone. To avoid the effects of metabolites, the incubation medium and tested extracts were changed for fresh ones every 24 h. Time dependent effects were examined after 24, 48 and 72 h cell treatments.

### 3.5. Cell Proliferation Test

Viability of cells in monolayers was assessed by the Proliferation reagent WST-1 (Roche, Mannheim, Germany) a ready-to-use colorimetric assay for the non-radioactive quantification of cellular proliferation. In a typical experiment cells after treatment with the tested samples were washed with PBS and incubated with the reagent solution (1:10 diluted with PBS) for 4 h at 37 °C and 5% CO_2_. The colorimetric measurement of the dark red product was measured at 440 nm in a multi-well EPOCH plate reader spectrophotometer (BioTek Instruments, Inc., Winooski, VT, USA).

### 3.6. Calculations and Statistical Analysis

The effects of tested extracts on cell proliferation were calculated as the effect (%) of individual dose *vs* value for control (untreated cells) at each time point. The data are expressed as the means ± SE. Presented values are the result of three individual experiments performed in triplicate for each dose. The efficiency of each extract is expressed by ED_50_ (the effective dose inducing 50% effect) and by the magnitude of maximal effect, e.g., the % of survived cells. The ED_50_ values and maximal effects were calculated from dose response curves using the computer program GraphPad Prism (GraphPad Software, Inc., La Jolla, San Diego, CA, USA). Statistical differences between time intervals and individual extracts were evaluated by one-way ANOVA and Bonferroni *post hoc* test.

## 4. Conclusions

Water extracts (lyophilisates) of selected *Cornus* species leaves were tested for their *in vitro* antiproliferative activity on human breast carcinoma MCF-7 cells. The present results demonstrated the dose- and time-dependent antiproliferative effects of all tested extracts. The most potent were those obtained from the CO, CM and CA species. Further, the antiproliferative activity results were correlated with polar secondary metabolites content. A negative correlation of total polyphenols and tannins with antiproliferative activity was proven. Furthermore, a positive correlation of biological activity with flavonoids and hydroxycinnamic acid derivative contents suggests the protective role of these substances on cells’ viability. The promising results of the present study demand further detailed studies with other carcinoma cells focused on the intracellular mechanism(s) of the cell growth inhibitory action.
